# Adapting psychiatric research in the age of COVID-19: role of online studies

**DOI:** 10.1192/bjo.2021.1015

**Published:** 2021-09-24

**Authors:** Andrew M. Novick, Joel Stoddard, Rachel L. Johnson, Mary D. Sammel, Lily Berkowitz, C. Neill Epperson

**Affiliations:** Department of Psychiatry, University of Colorado-Anschutz Medical Campus, USA; Department of Biostatistics and Informatics, Colorado School of Public Health, University of Colorado-Anschutz Medical Campus, USA; Department of Psychiatry, University of Colorado-Anschutz Medical Campus, USA; and Department of Biostatistics and Informatics, Colorado School of Public Health, University of Colorado-Anschutz Medical Campus, USA; Department of Psychiatry, University of Colorado-Anschutz Medical Campus, USA; Department of Psychiatry, University of Colorado-Anschutz Medical Campus, USA; and Department of Family Medicine, University of Colorado-Anschutz Medical Campus, USA

**Keywords:** COVID-19, online research

## Abstract

Virtual platforms can provide a socially distanced mechanism by which to promote ongoing research progress in the coronavirus disease 2019 (COVID-19) era and may change our approach to online research in the future. Understanding how to best utilise online research represents an important task for our field.

Although the coronavirus disease 2019 (COVID-19) pandemic has prompted an increase in research related to the SARs-COV-2 virus itself, it simultaneously has placed significant barriers on research in other areas. In mid-March 2020 many institutions, including the University of Colorado-Anschutz Medical Campus, halted most clinical research projects that did not have a clear benefit to human participants. Although research with human participants has resumed at many institutions under carefully orchestrated ‘ramp ups’, the disruption has been significant.

The disruption in research activity has been particularly detrimental to early career investigators in psychiatry,^1^ for whom progress with research is essential to establishing a track record of productivity and independence from one's mentors. Women in academia are especially hard-hit by the pandemic as evidenced by a decrease in female-authored publications since March 2020.^2^ Acquiring data and moving one's research programme forward requires adaptations of protocols and tools for data collection that allow for flexibility to balance the serious challenges imposed by the pandemic.

Prior to March 2020 and institution closure to all but life-preserving research, our group was investigating the effects of oral contraceptive pills on reward processing in women. By early April 2020, it became obvious that our plans to do in-person assessments, including neuroimaging during a reward task, would no longer be viable for an unforeseen time. Instead of delaying our investigation, we pursued online research methods that did not require in-person contact and that would further advance our knowledge regarding steroid contraceptive effects on reward. In sharing our experience with other researchers,^3^ we were met with considerable interest and curiosity. This suggested that the psychiatric community would benefit from learning more about conducting online research and evaluating its role even beyond the pandemic. Of course, online research in psychiatry is not necessarily new: literature on the implementation and evaluation of online psychological interventions dates back over 20 years.^4^ Thus, although much has been written about online research,^5,6^ here we aim to give a brief description of our experience and discuss the advantages of online research, available tools and potential limitations, such that others might consider its implementation.

## Online study design and recruitment

For those interested in exploring online research methods, Sauter et al^6^ recently published a brief yet thorough guide to the necessary steps, tools and considerations for running online behavioural experiments. We designed our study in REDCap,^7^ a platform for research database management, which allows for participant-facing surveys and task participation via the web. Importantly, we adapted the reward task that was originally planned for use during functional magnetic resonance scanning. This task sought to measure both reward anticipation and initial response to reward for various types of images (erotic images, non-erotic pleasant images such as puppies and scenery, as well as neutral images such as furniture). REDCap's interface was well suited for this task, allowing for stimulus presentation along with user input of image ratings. A number of platforms for delivering novel, online tasks are available – from extensions of lab-based software such as PsychoPy and E-Prime to online-only platforms such as Testable – REDCap was sufficient for our needs and we were already familiar with using it. A list of various resources that can assist in online study design and implementation can be found in the Appendix.

Recruitment of participants occurred through various internet media, including university listservs, social media and the use of established platforms, such as ResearchMatch that connect researchers with potential volunteers and allow targeted advertising to specific volunteers (Appendix). Over a period 2.5 months, we recruited *n* = 1000 eligible participants, half were current users of hormonal contraceptives and half who did not use hormonal contraceptives. This provided us with a large data-set to apply multivariable statistical and reinforcement learning models in order to evaluate the relationship between contraception use and reward processing. Furthermore, the data-set serves as preliminary results that will hone our original hypotheses and design of an in-person, neuroimaging experiment with the most robust stimuli possible to distinguish differences between groups.

## Advantages and limitations of online research

We found that one of the biggest advantages of online research is that it removes geographic barriers allowing researchers to recruit participants nationally and internationally as well as individuals from more isolated regions. The ability to complete studies from home may also eliminate issues of stigma that prevent individuals participating in research.^8^ It also allows for the rapid accrual of large amounts of data. And because it requires minimal equipment and less investment on the part of the participant in terms of time and travel, it is also an economic research strategy that can be used by early career investigators and others who have yet to acquire significant funding.

Although online research has the obvious benefit of feasibility during the pandemic, it has known limitations and disadvantages.^8^ The requirement for internet access and necessary computer skills is biased against low-income individuals with less education.^9^ As all eligibility information is gathered by self-report with minimal interaction with research staff, there is increased risk of inadvertent erroneous reporting or frank deceptive practices. Some individuals may rush through study questions with little thought to their responses whereas others may even set up automated methods or ‘bots’ in order to redeem payment with minimal effort. These risks can be minimised by putting in quality control measures, such as using online CAPTCHA (Completely Automated Public Turing test to tell Computers and Humans Apart) tools and interspersing questions that deliberately ask for a certain response (i.e. ‘Please select option 4’).

Even with quality assurance strategies, there are differences in the environment in an online versus lab setting for cognitive tasking that need to be better understood. Whereas one participant might be participating on their computer in a quiet environment, another might be on their phone while watching television. Such variation might be particularly relevant when asking participants to complete tasks with greater cognitive demands.^10^ However, researchers considering online experiments should be reassured by data suggesting that attention to tasks^11^ and data quality^12,13^ are similar between online and lab participants. Furthermore, anonymous, online tasking might promote more naturalistic behaviour, when the respondent is less influenced by an unfamiliar environment or the experimenter. The latter is known to lead to social desirability or demand effects, which may be particularly important in experiments like ours, in which we ask individuals to rate their experience of erotica.^14^ In any event, as the pandemic has promoted a shift for online clinical and research assessment, our field will need to better understand its implications for study design, analysis and interpretation. Thus, ongoing comparisons of similar methods that are conducted in laboratory environments versus at home via the internet will be necessary.

## Conclusion

The COVID-19 pandemic has created both challenges and opportunities for psychiatric research. Here we described how utilising an online research strategy enabled data collection and progress to inform our future work when other methods were not feasible. As opposed to a replacement for laboratory-controlled experiments, we discovered how online research could complement and inform our in-person experimental designs. During this time of unpredictability, we suggest this to others as a strategy for ongoing research progress. As patients and clinicians are adapting towards a responsible use of telehealth that will outlast the pandemic, so too should clinical researchers shift towards a better understanding and use of online sampling.

## Appendix

### Example resources available for conducting online studies

**Table:**

Resource	Category	Web address
REDCap	Research database and survey design and management	http://www.project-redcap.org
ResearchMatch	Participant recruitment	http://www.researchmatch.org
Amazon Mechanical Turk (mTURK)	Participant recruitment	http://www.mturk.com
Prolific	Participant recruitment	http://www.prolific.co
PsychoPy	Task/experiment design	http://www.psychopy.org
E-Prime	Task/experiment design	http://pstnet.com
Testable	Task/experiment design and participant recruitment	http://www.testable.org
Gorrilla	Task/experiment design and hosting	http://gorilla.sc
Qualtrics	Survey design and hosting	http://qualtrics.com
Survey Monkey	Survey design and hosting	http://www.surveymonkey.com

A more comprehensive list can be found in Sauter et al^6^
